# Plant Dynamic Metabolic Response to Bacteriophage Treatment After *Xanthomonas campestris* pv. *campestris* Infection

**DOI:** 10.3389/fmicb.2020.00732

**Published:** 2020-04-22

**Authors:** Marina Papaianni, Debora Paris, Sheridan L. Woo, Andrea Fulgione, Maria Manuela Rigano, Ermenegilda Parrilli, Maria L. Tutino, Roberta Marra, Gelsomina Manganiello, Angela Casillo, Antonio Limone, Astolfo Zoina, Andrea Motta, Matteo Lorito, Rosanna Capparelli

**Affiliations:** ^1^Department of Agricultural Sciences, University of Naples Federico II, Naples, Italy; ^2^Institute of Biomolecular Chemistry, National Research Council, Naples, Italy; ^3^Department of Pharmacy, University of Naples Federico II, Naples, Italy; ^4^Task Force on Microbiome Studies, University of Naples Federico II, Naples, Italy; ^5^Istituto Zooprofilattico Sperimentale del Mezzogiorno, Naples, Italy; ^6^Department of Chemical Sciences, University of Naples Federico II, Naples, Italy; ^7^Institute for Sustainable Plant Protection, National Research Council, Naples, Italy

**Keywords:** *Xanthomonas campestris* pv. *campestris*, bacteriophages, plant infection, metabolic response, gene expression

## Abstract

Periodic epidemics of black rot disease occur worldwide causing substantial yield losses. *Xanthomonas campestris* pv. *campestris* (*Xcc*) represents one of the most common bacteria able to cause the above disease in cruciferous plants such as broccoli, cabbage, cauliflower, and *Arabidopsis thaliana*. In agriculture, several strategies are being developed to contain the *Xanthomona*s infection. The use of bacteriophages could represent a valid and efficient approach to overcome this widespread phenomenon. Several studies have highlighted the potential usefulness of implementing phage therapy to control plant diseases as well as *Xcc* infection. In the present study, we characterized the effect of a lytic phage on the plant *Brassica oleracea* var. *gongylodes* infected with *Xcc* and, for the first time, the correlated plant metabolic response. The results highlighted the potential benefits of bacteriophages: reduction of bacterium proliferation, alteration of the biofilm structure and/or modulation of the plant metabolism and defense response.

## Introduction

*Xanthomonas campestris* pv. *campestris* (*Xcc*) is an economically important bacterial plant pathogen worldwide causing black rot disease that devastates many cultivated cruciferous crops, producing V-shaped necrotic lesions on the foliar margins and blackened veins (Alvarez, 2000). *Xcc* lives epiphytically on the leaf surface, infects the host penetrating stomas, hydathodes or wounds, and colonizes the vascular system of many Brassicaceae, including broccoli, cabbage, cauliflower, radish, and the model plant *Arabidopsis thaliana* (Danhorn and Fuqua, 2007). *Xcc* infection is particularly harmful due to the formation of biofilm, which contains degradative extracellular enzymes and other virulence factors (Dow et al., 2003).

Plants have developed different defense mechanisms against pathogens (Bostock et al., 2014). They respond to “pathogen associated molecular patterns” (PAMPs) by activating a PAMP-triggered immunity (PTI) or an effector-triggered immunity (ETI) mediated by receptors able to specifically recognize pathogens (Boller and He, 2009; Navarova et al., 2012). The consequence can be the establishment of a “systemic acquired resistance” (SAR) status, which may increase resistance in the whole plant to subsequent attacks (Navarova et al., 2012; Shah et al., 2014; Schwachtje et al., 2018).

Defense responses have metabolic costs in terms of energy and resources, normally used to support processes of development and reproduction (Schleucher et al., 1994). Indeed, during pathogen infection, photosynthesis is down regulated, as a result of primary metabolism reorganization. Transcriptional analysis confirms that the metabolic reprogramming caused by pathogen infection is mainly associated with genetic and biochemical changes in basic pathways, such as those involved in the synthesis or degradation of carbohydrates, amino acids, and lipids, as well as in defense response (Rojas et al., 2014). Contact with the pathogen often causes up-regulation of genes involved in energy production processes, such as glycolysis, the pentose phosphate pathway, Krebs cycle, mitochondrial electron transport, ATP, and amino acid biosynthesis (Brauc et al., 2011).

In agriculture, crop protection strategies based on beneficial microorganisms or naturally-derived antimicrobial agents are being developed in order to reduce the impact on non-target organisms, including humans (Kang et al., 2018). To this end, bacteriophages (phages) may be particularly useful. They self-replicate only as long as the bacterial host is present, which may reduce the need of multiple applications (Sabouri Ghannad and Mohammadi, 2012). Moreover, phages – being considered the most common biological entities on earth (Papaianni et al., 2018) – can be found in a variety of forms and environments (Koskella and Meaden, 2013). They are non-toxic for eukaryotic cells, and, due to their specificity, may not harm the soil beneficial microbiota (Capparelli et al., 2010; Reddy, 2013; Górski et al., 2018).

Several studies have stressed the potential usefulness of implementing phage therapy to control plant diseases (Svircev et al., 2018). This is the case also of *Xcc*, for which different research groups have isolated specific phages (Weiss et al., 1994). However, the use of phage therapy in plants is still poorly studied.

Metabolomics is particularly apt to investigate plant-pathogen interactions, and to understand the mechanisms of innate immunity (Hagemeier et al., 2001; Desbrosses et al., 2005; Lindon et al., 2005). High-resolution NMR spectroscopy and multivariate data analysis have been widely used in order to evaluate the occurring changes based on a holistic approach (Saviano et al., 2016). However, to date, the metabolic impact of phage-bacterial infections on the plant have not yet been described.

In the present study, we isolated and characterized a lytic bacteriophage (Xccφ1) able to control the disease caused by *Xcc* to *Brassica oleracea* var. *gongylodes* and investigated the effect of *Xcc* and Xccφ1, applied singly or combined, on plant metabolome. Finally, our results indicate that phage-based treatments limit the bacterium proliferation, due to lysis of the host, alteration of the biofilm structure and/or modulation of plant defense response.

## Materials and Methods

### Isolation of *Xanthomonas campestris* pv. *campestris*

Leaves of cauliflower and kohlrabi plants with symptoms of black rot were collected from cultivated crops in Campania (Piana del Sele, Italy) during January–February 2017. After a thorough washing with sterile distilled water, tissue fragments were ground in sterile distilled water (SDW) and streaked on mCS20ABN agar medium (Schaad et al., 2003). Yellow mucoid colonies were extensively purified on Nutrient Agar (Sigma Aldrich, Milan, Italy) supplemented with glucose 0.5% (NAG). Single colonies were then suspended in SDW and stored at 4°C. The isolates were identified by the Biolog^TM^ System (Hayward, CA, United States) as *Xcc*. The Biolog identified strains were tested for their sensitivity to the phage, and inoculated by spraying (1 × 10^7^ CFU/mL in sterile distilled water) on kohlrabi plantlets to verify their virulence. After 12 days symptoms were evaluated and the most virulent isolate (*Xcc* number 7) was selected and used in the next experiments.

### Species-Specific PCR of *Xcc*

Molecular diagnosis of *Xcc* was carried out using the primers HrcCF2 (5′-CGTGTGGATGT GCAGACC-3′) and HrcCR2 (5′-CAGATCTGTCT GATCGGTGTCG-3′), which amplify an internal fragment of 519 bp of hrcC (Zaccardelli et al., 2007).

### Morphological Characterization of *Xcc*

Curli and cellulose productions were detected by growing bacteria on Nutrient Agar supplemented with Congo-red (4 mg/mL Sigma Aldrich) and on Nutrient Agar supplemented with calcofluor (10 mg/mL Sigma Aldrich), respectively. Plates were incubated at 24–25°C for 72 h. Calcofluor colonies were visualized under a 366-nm light source (Barak et al., 2005).

### Isolation and Growth of *Xcc* Phages

Ten grams of rhizospheric soil of 100 cauliflower and kohlrabi plants with Black rot symptoms were suspended in 15 mL of Nutrient broth (Sigma Aldrich), and agitated for 30 min at 20°C. Soil sediments were removed by centrifugation, and the supernatants transferred to sterile flasks. Log-phase cultures of *Xcc* were added and flasks incubated overnight at 24°C in shaking condition. Cultures were clarified by centrifugation and filtered through a Millipore 0.22 μm-pore-size membrane filter (MF-Millipore, Darmstadt, Germany). The filtrates were assayed for the presence of *Xcc*-infecting phage by plating (10 μl) on soft agar overlay for 48 h. The clear plaque on soft-agar containing phage were picked and incubated for 4 h at 37°C, centrifuged for 30 min at 5000 rpm and filtered through 0.22-μm-pore-size membrane filters (Cross et al., 2015). The experiment was performed for 5 time. At least, suspensions were stored at 4°C.

### Host Range Analysis

The lytic activity of all the isolated phages (Xccφ1) was tested on 23 different *Xanthomonas* isolates (Supplementary Table S1). Individual *Xanthomonas* strains grown in NB to the exponential phase were added (500 μl) individually to tubes containing 4 mL of 0.7% agar (Sigma Aldrich, Milan, Italy). The suspension was transferred to a Petri dish with nutrient agar and let to solidify. 10 μl of all the phage were spotted on agar plates, which were then incubated at 25°C for 48 h (Garbe et al., 2010). The experiment was performed in triplicate.

### Multiplicity of Infection

The ratio between virus particles and host cells was used to determine the Multiplicity of infection (MOI; Jacobs et al., 1993). The *Xcc* strain was grown in Nutrient Broth (NB) at 24°C to the concentration of 10^8^ CFU/mL, as determined by measuring the optical density at 600 nm. Cells at the exponential growth phase, were infected with phage (10^3^ to 10^–3^ PFU/CFU) in a 96 well plates (Corning^®^ 96 Well CellBIND^®^ Microplates, Sigma Aldrich), then incubated at 24°C for 48 h. CFUs were counted by standard soft agar overlay assay (Klement et al., 1990; Fulgione et al., 2019). Experiments were performed in triplicate. Optimal MOI, that resulted in the highest phage titer within 48 h incubation, was used in subsequent phage propagation.

### Burst Size Analysis

1 mL of exponential-growth-phase culture of *Xcc* in NB (10^8^ CFU/mL) and phage suspension were mixed at MOI of 0.1. The mixture was incubated at 24°C for 5 min to allow phage adsorption. Immediately after, the mixture was diluted to 10^–4^ in 50 mL Erlenmeyer flasks. Samples were taken from the diluted fraction at ten-min intervals, serially diluted ten-fold and spotted on NA plates using the agar overlay technique. The experiment was repeated three times. The latent period was expressed as the time interval between phage adsorption (which does not include the 5-min pre-incubation time) and the first burst (Drulis-Kawa et al., 2011; Rigvava et al., 2013). Burst size was calculated as the ratio between the final count of liberated phage particles and the initial count time of infected bacterial cells during the latent period.

### pH Stability

The phage stability at different pH was assessed using the double-layer agar technique. The pH of SM buffer was adjusted to the following values using 1 M NaOH or 1 M HCl: pH 1 to 11 (Jepson and March, 2004). Subsequently, the plates were incubated at 25°C for 48 h. The lysis spots were picked and inoculated into 500 μl of buffer SM solutions at different pH and incubated at 37°C for 4 h. The solutions were centrifuged at 5000 rpm at room temperature for 30 min, filtered through 0.22 μm filters (MF-Millipore), and incubated at RT for 7 days. 10-fold dilutions of each solution were spotted (10 μl) on the agar plate. The plates were incubated at 25°C for 48 h and evaluated the final concentration of each condition.

### Phage Purification

To purify Xccφ1, 1 mL of the lysate was centrifuged at 14 K RPM for 2 h at room temperature, then the supernatant was discarded and 200 μl of 5 mM MgSO_4_ (Sigma Aldrich) was added, and the pellet was incubated overnight at 5°C. The pellet was resuspended by gently pipetting up and down, diluted 2× and 4× in 5 mM MgSO_4_.

### Transmission Electron Microscopic Analysis

The Xccφ1 stock (10^8^ PFU/mL) was purified by CsCl density gradient ultracentrifugation (Centrifuge for 2.5 h 24 K in the SW 28.1) and dialyzed against SM buffer overnight at 4°C. Phage particles were negatively stained with 2% phosphotungstic acid (pH 7.2) for 5 min. Phages were observed in a Philips EM 300 electron microscope.

### Chemical Analysis

Glycosyl analysis was performed as reported by Fresno et al. (2007) and Casillo et al. (2017b).

### Confocal Laser Scanning Microscopy

Biofilms were formed on polystyrene Nunc^TM^ Lab-Tek^®^ 8-well Chamber Slides (n°177445; Thermo Scientific, Ottawa, ON, Canada). For this purpose, overnight cultures of *Xcc* in Nutrient broth were diluted to a cell concentration of about 0.001 (OD_600 nm_) and inoculated into each well of a chamber slide. The bacterial culture was incubated at 24°C for 72 h in order to assess the biofilm thickness and cell viability. After 10^6^ and 10^8^ PFU/mL of phage were added for 6 h. The biofilm cell viability was determined with the FilmTracer^TM^ LIVE/DEAD^®^ Biofilm Viability Kit (Molecular Probes, Invitrogen, Carlsbad, CA, United States) according to Papaianni et al. (2018).

### Static Biofilm Analysis

Biofilm formation was monitored using the Christal violet assay. *Xcc* bacteria were incubated for 72 h in NB at 24°C and after the biofilm formation the galactose was added at different concentrations (from 0.5 to 2%) and incubated for 4 h at 24°C. The biofilm was analyzed at 590 nm after the staining with Crystal violet (Sigma Aldrich; Merritt et al., 2005).

### In-Planta Experiments

Seeds of *B. oleracea* var. *gongylodes* – susceptible to the disease – were sown in 60-well Styrofoam planting trays containing steamed sterile soil peat mixture. The trays were kept for 48 h in a germination chamber and then transferred to a glasshouse. All the experiments were carried out with a temperature of 15 ± 2°C (night) and 25 ± 2°C (day). At the stage of the second true leaf, the plantlets were used for the experiments. The *Xcc* strain was grown on NAG Petri dishes for 36 h at 28°C and the bacterial growth suspended in SDW. The final bacterial concentration was spectrophotometrically adjusted to the established level. Seedlings were treated using a hand-held plastic sprayer with SDW and suspensions of phage and *Xcc* supplemented with Tween 20 (5 μl per 100 mL). Two experiments were conducted. The first trial was performed to determine if the timing of phage application had any influence on the pathogenic activity of *Xcc* when the bacterium was inoculated on a host plant. Kohlrabi (*B. oleracea* var. *gongylodes)* plantlets were treated as follows: (a) SDW, (b) Xccφ1, (c) *Xcc*, (d) Xccφ1 24 h before inoculation with *Xcc*, (e) Xccφ1 and *Xcc* together, (f) Xccφ1 24 h after *Xcc*, and (g) Xccφ1 48 h after *Xcc*. Both phage and bacterium were suspended in SDW at 10^7^ PFU/mL and 10^7^ CFU/mL, respectively.

The second trial was performed to determine if the concentration of the phage application influenced the pathogenic activity of *Xcc;* the plantlets were treated as follows: (a) SDW, (b) Xccφ1 10^9^ PFU/mL, (c) *Xcc* 10^8^ CFU/mL, and (d) Xccφ1 10^9^ PFU/mL and *Xcc* 10^8^ CFU/mL together. Trials were planned according to a randomized block design with three replications for each treatment. Each replication was made up of one tray with 60 plantlets. After inoculation, the plantlets were kept under clear plastic storage boxes, irrigated daily, and misted with distilled water twice a day to maintain a high level of relative humidity to aid infection by the pathogen. 15 days after inoculation, infection symptoms were rated according to a four-level arbitrary disease scale whereby: 0 (no symptoms) to 3 (all leaves with symptoms and/or strong defoliation).

The empirical scale allowed the calculation of McKinney’s index, expressed both as the weighted average of the disease and as a percentage of the maximum possible level (McKinney, 1925). The non-transformed values of the McKinney indexes were submitted to analysis of variance (ANOVA) and the significance of the differences was calculated by Tukey’s test (*p* < 0.05).

### RNA Extraction and Expression Profiling by qPCR

Plantlets treated as described in the second trial of the *in planta*-experiments were used to analyze the expression profiling of genes involved in (1) synthesis/degradation of GABA at 15 days post-inoculation (dpi) or (2) in disease resistance at 48 h post inoculation (hpi). Plants were washed with SDW and immediately frozen in liquid nitrogen. Total RNA was extracted and purified using PureLink^®^ RNA Mini Kit (Ambion Inc., Austin, TX, United States) from a pool of equal amounts of the powdered plant tissue obtained from 3 biological replicates for each treatment. Removal of genomic DNA was performed by digestion with DNase I, Amplification Grade (Invitrogen, United States). The Qubit^*rmTM*^ RNA BR Assay Kit and Qubit^*rmTM*^ 2.0 Fluorometer (Life Technologies, Thermo Fisher Scientific Inc., Denver, CO, United States) were used to assess total RNA quantity, while the quality was verified by NanoDrop^®^ ND-1000 (Thermo Fisher Scientific Inc.). Only RNA samples with 230/260 and 260/280 ratios > 2 were used in the further analyses. 1 μg of purified total RNA was used as a template for first-strand cDNA synthesis using SuperScript^®^ III Reverse Transcriptase (Invitrogen). Gene transcript levels were measured using Power SYBR^®^ Green PCR Master Mix (Applied Biosystems^®^) on a QuantStudio 3 Real-Time PCR System (Applied Biosystems^®^, Thermo Fisher Scientific^TM^, Waltham, MA, United States) with the following conditions: an initial step at 95°C for 10 min, followed by 45 cycles of 95°C for 10 s, 60°C for 20 s and 72°C for 10 s. QuantStudio Design and Analysis Sofware v1.1 (Applied Biosystems) was used for analysis of gene expression. All samples were normalized to actin as reference housekeeping gene. The relative quantitative expression was determined using the 2^–ΔΔCT^ method (Livak and Schmittgen, 2001). All primers used in this work are reported in Supplementary Table S3 (Kim et al., 2013b; Faës et al., 2015).

### Extraction Procedure and Sample Preparation for NMR

A total of different 40 samples were used, each class (NT, *Xcc*, Xccφ1, and *Xcc* + Xccφ1) containing 10 samples. To extract the metabolites of interest (e.g., lipids, carbohydrates, amino acids, and other small metabolites), while eliminating DNA, RNA, and proteins, tissues were mechanically disrupted. Combined extraction of polar and lipophilic metabolites was carried out by using a methanol/chloroform protocol (Lindon et al., 2005). 0.5 g/plant of frozen vegetal tissue were powdered in a ceramic mortar with a pestle. Tissues were transferred in centrifuge tubes and 4 mL of methanol, 1.70 mL of water, and 4 mL of chloroform per gram of wet tissue (all solvents were cold) were added to the tube, and vortexed for 30 s. The sample was gently stirred and mixed, on ice, for 10 min (the solution must be mono-phasic). 4 mL of chloroform and 4 mL of water per gram of wet tissue were added, and the final mixture was vortexed and centrifuged at 3000 rpm for 15 min at 4°C. This procedure separates three phases: water/methanol at the top (aqueous phase, with the polar metabolites), denatured proteins and cellular debris in the middle, and chloroform at the bottom (lipid phase, with lipophilic metabolites). The methanol/water and chloroform fractions were separately collected in 5-mL glass vials, dried in vacuum at room temperature and stored at −80°C until required.

Prior to NMR analysis, the methanol/water fractions were resuspended in 630 μl of phosphate buffer saline (PBS, pH 7.2), and 70 mL of a deuterated-water solution (containing 1 mM sodium 3-trimethylsilyl [2,2,3,3-^2^H_4_] propionate (TSP) as a chemical shift reference for^1^H spectra). The deuterated solvent was added to provide a field- frequency lock so that each sample reached 700 mL of total volume into the NMR tubes.

### NMR Analysis

One-dimensional (1D) spectra were recorded on a Bruker Avance III–600 MHz spectrometer (Bruker BioSpin GmbH, Rheinstetten, Germany), equipped with a TCI CryoProbe^TM^ fitted with a gradient along the *Z*-axis, at a probe temperature of 27°C. One-dimensional (1D) proton spectra were acquired at 600 MHz by using the excitation sculpting sequence (Hwang and Shaka, 1995). A double-pulsed field gradient echo was used, with a soft square pulse of 4 ms at the water resonance frequency, with the gradient pulse of 1 ms each in duration, adding 516 transients of 16384 points with a spectral width of 8417.5 Hz. Time-domain data were all zero-filled to 32768 points, and prior to Fourier transformation, an exponential multiplication of 0.8 Hz was applied. For two-dimensional (2D) clean total correlation spectroscopy (TOCSY; Bax and Davis, 1985; Griesinger et al., 1988) spectra we used a standard pulse sequence with a spin-lock period of 64 ms, achieved with the MLEV–17 pulse sequence, and incorporating the excitation sculpting sequence for water suppression. In general, 256 equally spaced evolution-time period *t*_1_ values were acquired, averaging 64 transients of 2048 points, with 8403.36 Hz of spectral width. Time-domain data matrices were all zero-filled to 4096 points in both dimensions, thus yielding a digital resolution of 2.04 Hz/pt. Prior to Fourier transformation, a Lorentz-to-Gauss window with different parameters was applied for both *t*_1_ and *t*_2_ dimensions for all the experiments. Spectra in water were referred to internal 0.1 mM TSP, assumed to resonate at δ = 0.00 ppm. Natural abundance 2D ^1^H–^13^C heteronuclear single quantum coherence (HSQC) spectra were recorded at 150.90 MHz for ^13^C, using an echo-antiecho phase sensitive pulse sequence with adiabatic pulses for decoupling (Kay et al., 1992; Lee et al., 2014) and pre- saturation for water suppression (Schleucher et al., 1994). 128 equally spaced evolution time period *t*_1_ values were acquired, averaging 240 transients of 2048 points, and using GARP4 for decoupling. The final data matrix was zero-filled to 4096 in both dimensions, and apodized before Fourier transformation by a shifted cosine window function in *t*_2_ and in *t*_1_. Linear prediction was also applied to extend the data to twice its length in *t*_1_. HSQC spectra in water were referred to the α-glucose doublet resonating at 5.24 ppm for ^1^H and 93.10 ppm for ^13^C.

### Multivariate Data Analysis

The 0.70–9.70 ppm spectral region of aqueous extracts was automatically data reduced to integrated regions (buckets) of 0.02-ppm width using the AMIX 3.9.7 package (Bruker Biospin GmbH). The residual water resonance region (4.50–5.06 ppm) was excluded, and each integrated region was normalized to the total spectrum area. To discriminate samples using NMR profiles, a multivariate statistical data analysis was carried out using projection methods. The matrix of the integrated data was imported into SIMCA14 package (Umetrics, Umeå, Sweden) and Principal Component Analysis (PCA) and Orthogonal Projection to Latent Structures Discriminant Analysis (OPLS–DA) were performed. Unit variance scaling was used as data pre-treatment for both PCA and OPLS–DA. PCA was first applied as unsupervised strategy to identify data trends. Next, OPLS-DA was used to better define clustering and relate metabolic variations to pathophysiological changes (Trygg and Wold, 2002). Validation of the models was carried out using 7-fold cross–validation and permutation tests (800 repeats) to verify possible model overfit. The quality of all PCA and OPLS–DA models was evaluated using the regression correlation coefficient *R*^2^ and the cross-validate correlation coefficient *Q*_2_. Normality test (Shapiro–Wilk and D’Agostino *K* squared) on normalized buckets of discriminant metabolites and non-parametric Kruskal–Wallis Anova test were performed with the OriginPro 9.1 software package (OriginLab Corporation, Northampton, United States). Moreover, for multiple comparisons, the Dunn Kruskal–Wallis test with Bonferroni correction was implemented in *R* (R Development Core, 2019)^1^, all the test results and the adjusted *p*-values are presented in supporting information material. Signal variations were presented as chemical shift assignments (Supplementary Table S2). Results were considered statistically significant at *p* < 0.05.

### Pathway Analysis

Pathway topology and biomarker analysis on selected and more representative discriminating metabolites were carried out using specific tools in Metaboanalyst 4.0 (Chong et al., 2018). We calculated the centrality through the Pathway Impact, a combination of the centrality and pathway enrichment results. Metabolites were selected by evaluating both VIP values > 1 in class discrimination and correlation values |pq[corr]| > 0.7. *A. thaliana* pathway library was chosen and analyzed using Fisher’s Exact Test for over representation and Relative- betweenness Centrality for pathway topology analysis.

## Results

### Isolation and Characterization of *Xcc*

**FIGURE 1:**
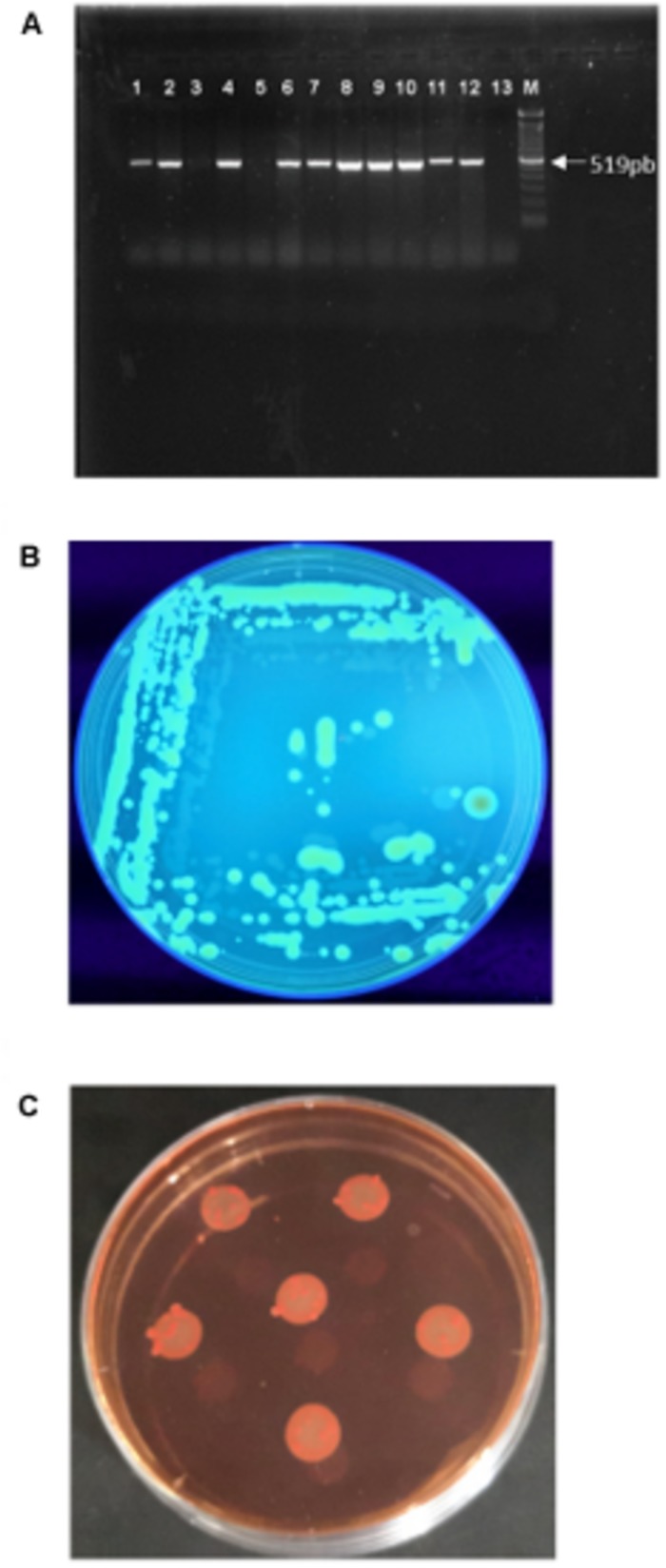
*Xanthomonas campestris* pv. *campestris* characterization: **(A)** Identification of the isolates by PCR amplification of the *Xcc* specific gene HrcC. Lane 1: positive control; Lanes 1–13: bacterial DNA; M: Marker (100 bp DNA Ladder). **(B)** Colony fluorescence on calcofluor agar plates due to cellulose synthesis. **(C)** Colonies on Congo red demonstrating the pdar phenotype due to the presence of fimbriae.

Twenty-seven bacterial isolates were obtained from *B. oleracea* plants displaying typical symptoms of *Xcc* infection and identified by PCR using *Xcc*-specific primers. Ten isolates resulted positive (Figure 1A) and were found to produce the main components of *Xanthomonas* biofilm (cellulose and curli; Figures 1B,C).

### Isolation and Characterization of Phage Xccφ1

**FIGURE 2:**
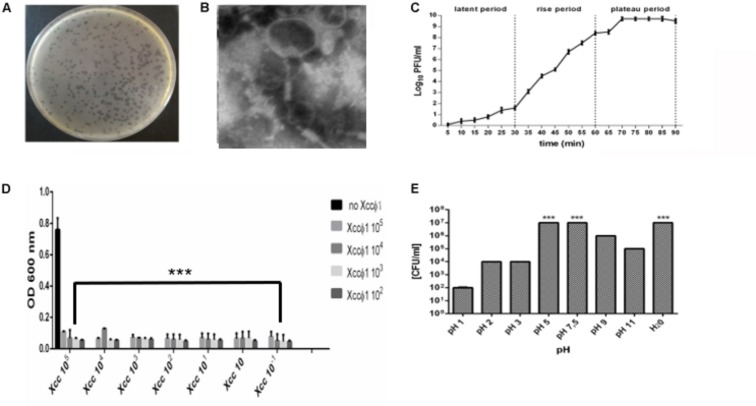
Xccφ1 characterization: **(A)** plaques on soft agar of approximately 2–3 mm in diameter. **(B)** Phage structure as observed by Transmission Electron Microscopy (TEM). Bar = 100 nm. **(C)** One step growth curve of phage. **(D)** Representation of phage activity on *Xcc* growth. The figure shows the final bacterial plate counts (CFU/mL) after the treatment with the phage and growth on Nutrient Agar. Each value is the mean ± SD of 3 independent experiments. **(E)** Phage stability in SM buffer at different pH and H_2_O control. ***, *p* < 0.001. Statistical analysis was performed with Student’s *t*-test. Values are the mean ± SD from 3 independent experiments with 3 replicates for each data point.

Phages were isolated from 17 soil samples obtained from the rhizosphere of *Brassica* plants. All phages displayed the same host range, were specific to *X. campestris* pathovar *campestris* only, forming clear plaques on all *Xcc* isolated from different area. Any phage isolated and tested are able to lyse the other *X. campestris* strains tested (Supplementary Table S1). On soft agar, Xccφ1 consistently formed clear plaques of approximately 2–3 mm in diameter (Figure 2A). Analysis by TEM revealed a structure typical of the *Myoviridae* family, with a contractile, long and relatively thick tail (120 × 30 nm), and a central core separated from the head by a neck (Figure 2B; King et al., 2011). Adsorption rate of Xccφ1 (27°C; 20 min) was 85, 70, and 65% at 10^5^, 10^6^, and 10^7^ PFU/mL, respectively. The latent period and burst size were 30 min and 42 ± 4 viral particles per infected cell, respectively, while the rise period was 30 min (Figure 2C). The phage growth curve displayed the canonical phases of latency, replication, and host lysis (Figure 2C). The lytic activity was phage concentration independent (Figure 2D). The host range of Xccφ1 included 12 bacterial isolates from Brassica plants (cauliflower, kohlrabi, and rocket). The concentration of the phage was not affected at pH 5 and 7.5 in SM buffer or in water (Figure 2E).

### Chemical Analysis

**FIGURE 3:**
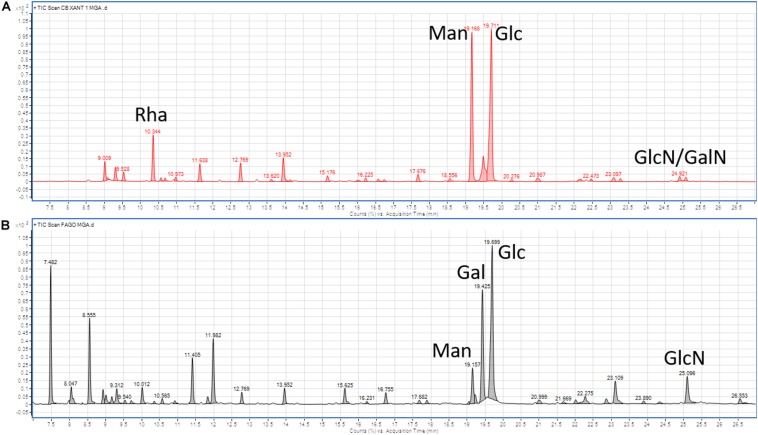
Gas chromatography-mass spectrometry (GC-MS) analysis of **(A)** *Xcc* cells in biofilm, and **(B)** Xccφ1 particles. Rha, Rhamnose; Man, mannose; Glc, glucose; GalN, galactosamine; GlcN, glucosamine; and Gal, galactose.

Glycosyl analysis of *Xcc* cells in biofilm (Figure 3A) revealed the presence of rhamnose (Rha), mannose (Man), glucose (Glc), and traces of galactosamine (GalN) and glucosamine (GlcN), all main components of exopolysaccharides (EPS; Casillo et al., 2017a). Phage analysis (Figure 3B) indicated the presence of Glc, galactose (Gal) and, at a lower concentration, Man, whereas the latter two are uncompetitive inhibitors of bacterial biofilm stability. These findings may suggest the role of galactose instead of mannose on biofilm maintenance and thus on the phage activity (Ryu et al., 2016).

### Confocal Laser Scanning Microscopy

**FIGURE 4:**
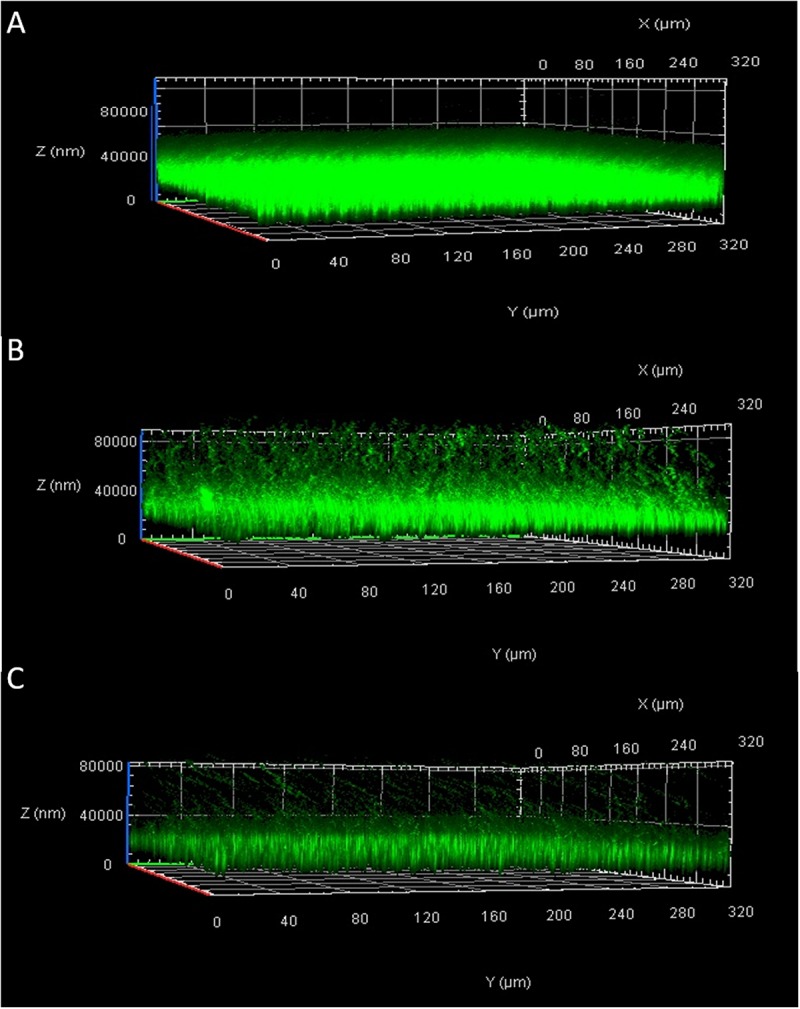
Effect of Xccφ1 phage concentration on the structure of *Xcc* biofilm as analyzed by Confocal Laser Scanning Microscopy (CLSM): **(A)**, *Xcc* alone; **(B)**, *Xcc* after 6 h of incubation with phage at 10^6^ PFU/mL; **(C)**, *Xcc* after 6 h of incubation with phage at 10^8^ PFU/mL. Bacteria were grown for 72 h in 8-well chamber slides and stained with LIVE/DEAD reagents. Green fluorescence (SYTO9) indicates viable and red fluorescence (PI) dead cells.

The biofilm analyzed by Confocal Laser Scanning Microscopy (CLSM) showed a structure that appeared thick and multi-layered in the absence of the phage and collapsed when bacteria were treated with Xccφ1 (Figure 4). The effect of the phage was concentration dependent, with a dose of 10^8^ PFU/mL added to the bacterial culture demonstrating a greater reduction in the structure of the biofilm in comparison to a dose of 10^6^ PFU/mL (cf. Figures 4B,C). The biofilm was also reduced by a treatment with galactose, a sugar present as a component of the Xccφ1 capsid (Figure 3B; Kaur et al., 2012), whereby different non-toxic concentrations (from 0.5% to 2%) were effective on the biofilm and 1.5 and 2% are significant (*p* < 0.001; Supplementary Figure S1). These findings indicate that the efficacy of biofilm disaggregation activity exerted by the phage may be supported by galactose, a result in line with a previous study (Doolittle et al., 1996).

### Phage Activity *in Planta*

**FIGURE 5:**
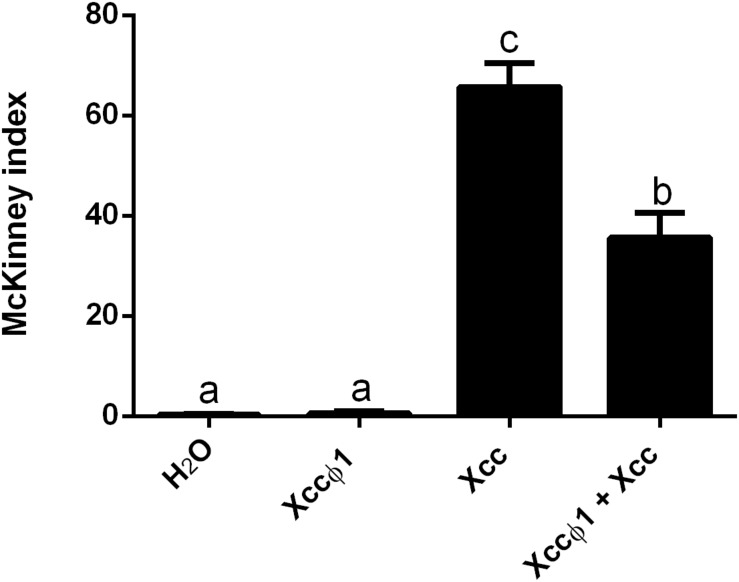
Effect of the Xccφ1 phage treatments on *Xcc* disease severity, as measured by the McKinney index, with foliar applications to plants of *B. oleracea* var. *gongylodes*. *Xcc* was inoculated at 10^8^ CFU/mL, while Xccφ1 at 10^9^ PFU/mL. In the combined treatment, phage, and bacterium were applied simultaneously. Values are the mean ± SD of three replicates (60 plantlets each) per treatment. Bars labeled with the same letter are not statistically different at the Tukey test (*p* < 0.05).

*Brassica oleracea* var. *gongylodes* plantlets were treated by spraying the aerial vegetative parts with suspensions of the phage and the bacterium at different times and concentrations. The results of the first trial showed a statistically significant decrease (20%) in disease symptoms on plants treated with the anticipated application of the phage 24 h before *Xcc* (Supplementary Figure S2). The effects on disease development were not significant when the phage and bacteria were sprayed together, at the same time. When the phage was applied 24 or 48 h after the *Xcc* inoculation, there was no disease control. In the second trial, Xccφ1 was applied together with *Xcc*, both at higher concentrations than those used previously (Xccφ1 at 10^9^ PFU/mL and *Xcc* at 10^8^ CFU/mL). In this case, the development of the disease symptoms was reduced by about 45% (Figure 5). Interestingly, in the glasshouse Xccφ1 survived and was detected on the plant leaf surfaces up to six weeks after application.

### NMR-Based Metabolomic Analysis

We acquired 92 ^1^H-NMR spectra from extracts (polar fraction) obtained from leaves of *B. oleracea* var. *gongylodes* receiving the phage and bacterium treatments conducted in the second trial. For each treatment, the most representative samples were analyzed by 2D NMR analysis. All resonances were identified by comparing 2D data with the literature and/or online databases (Supplementary Table S2). 1D-NMR metabolic profiles were subjected to multivariate statistical analysis in order to detect trends and clusters (Eriksson, 2006).

We tested the following leaf samples: 26 untreated (NT), 25 infected with *Xcc* (*Xcc*), 23 treated with *Xcc* plus the phage (*Xcc* + Xccφ1), and 18 treated with the phage alone (Xccφ1). Unsupervised PCA models (data not shown) displayed a clear clustering into four distinct groups that corresponded to the treatments and excluded the potential presence of outliers.

**FIGURE 6:**
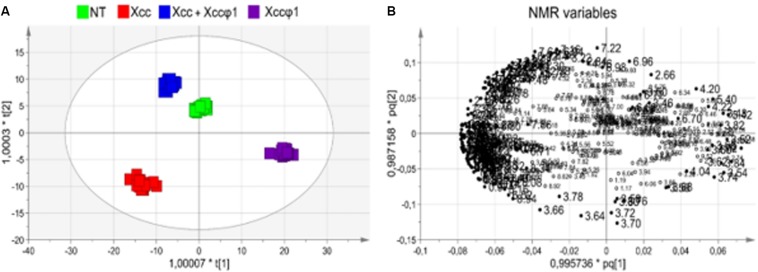
Metabolomic analysis (OPLS-DA of NMR data) of leaf extracts from *B. oleracea* var. *gongylodes* treated with *Xcc* and *Xcc* + Xccφ1. **(A)** Scores plot (97%, *p* < 0.0001) showing the separation of the treatments: NT (water); *Xcc* (bacteria alone); *Xcc* + Xccφ1 (*Xcc* plus Xccφ1 phage); Xccφ1 (phage alone). *R*^2^ was 0.97 and Q2 was 0.96. **(B)** Loadings plot associated with the OPLS-DA analysis reported in (A), indicating determining NMR variables. Numbers refer to buckets’ chemical shifts (spectral positions), and their size indicated the more discriminating buckets. The pq[1] and pq[2] values refer to the weight that combines the *X* and *Y* loadings (*p* and *q*).

OPLS-DA was applied to improve group separation. Regression analysis generated a robust model (*R*^2^ = 0.97, *Q*^2^ = 0.96) with three predictive components, and a clear separation in the scores plot (Figure 6A). In particular, the first component t[1] clearly differentiated the *Xcc* (red squares) and the *Xcc* + Xccφ1 groups (blue squares) from the phage Xccφ1 group (purple squares), with the NT group (green squares) located in the middle (Figure 6A). The second component t[2] discriminated between the *Xcc* and the *Xcc* + Xccφ1 groups, with the latter located very close to the control group (Figure 6A). The third component (t[3] on t[1]) differentiated the NT group along the t[3] positive axis (Supplementary Figure S3).

Hence, the projection of all samples along the combination of the first and the second components reflected the specific metabolic alterations among different groups, which cluster in specific areas of the statistical model. The *Xcc* and the Xccφ1 groups appeared in the III and the IV quadrants of the score plot, respectively, while the *Xcc* + Xccφ1 group is placed in the II quadrant, adjacent to the NT group (Figure 6A). This result indicates a small metabolic variation between healthy controls and leaves infected with both the phage and the bacterium. Therefore, the presence of Xccφ1 may have resulted in a significant variation of the disease-associated plant metabolome.

The loadings plot helped in the identification of NMR variables responsible for group separation (Figure 6B). We considered as discriminating only signals (bins) with variable influence on projection (VIP) values > 1 and |pq[corr]| > 0.7.

Statistically relevant biochemical information was obtained from discriminating metabolites in the OPLS-DA model, by using a univariate statistical analysis. Metabolite set enrichment analysis (MSEA) identified 30 major metabolic pathways involved and significantly modified processes in the plants. Among these: alanine, aspartate, and glutamate (*p* = 3.1 × 10^–5^, impact = 0.44); arginine and proline (*p* = 4.5 × 10^–3^, impact = 11); valine, leucine and isoleucine biosynthesis (*p* = 1.1 × 10^–2^, impact = 0.04); galactose (*p* = 1.1 × 10^–2^, impact = 0.05); lysine biosynthesis (*p* = 1.4 × 10^–2^, impact = 0.07); sucrose (*p* = 1.7 × 10^–2^, impact = 0.09), and glyoxylate and dicarboxylate (*p* = 3.8 × 10^–2^, impact = 0.27). The characteristics of the pathway are correlated with the size and color of the circles shown in Supplementary Figure S4. In particular, the relative size and the color (from yellow to red) of the circles indicates the pathway relevance for this study and the number of metabolites differentially produced and associated to a specific pathway. Although the alanine, aspartate, and glutamate metabolism (Holm *p* = 2.7 × 10^–3^, FDR = 1.3 × 10^–3^) appeared to be the most affected, all the pathways involved were considered and shown.

The water control (NT) and the three treatments (*Xcc*, *Xcc* + Xccφ1, and Xccφ1 alone) produced different changes in the plant metabolic profile.

**FIGURE 7:**
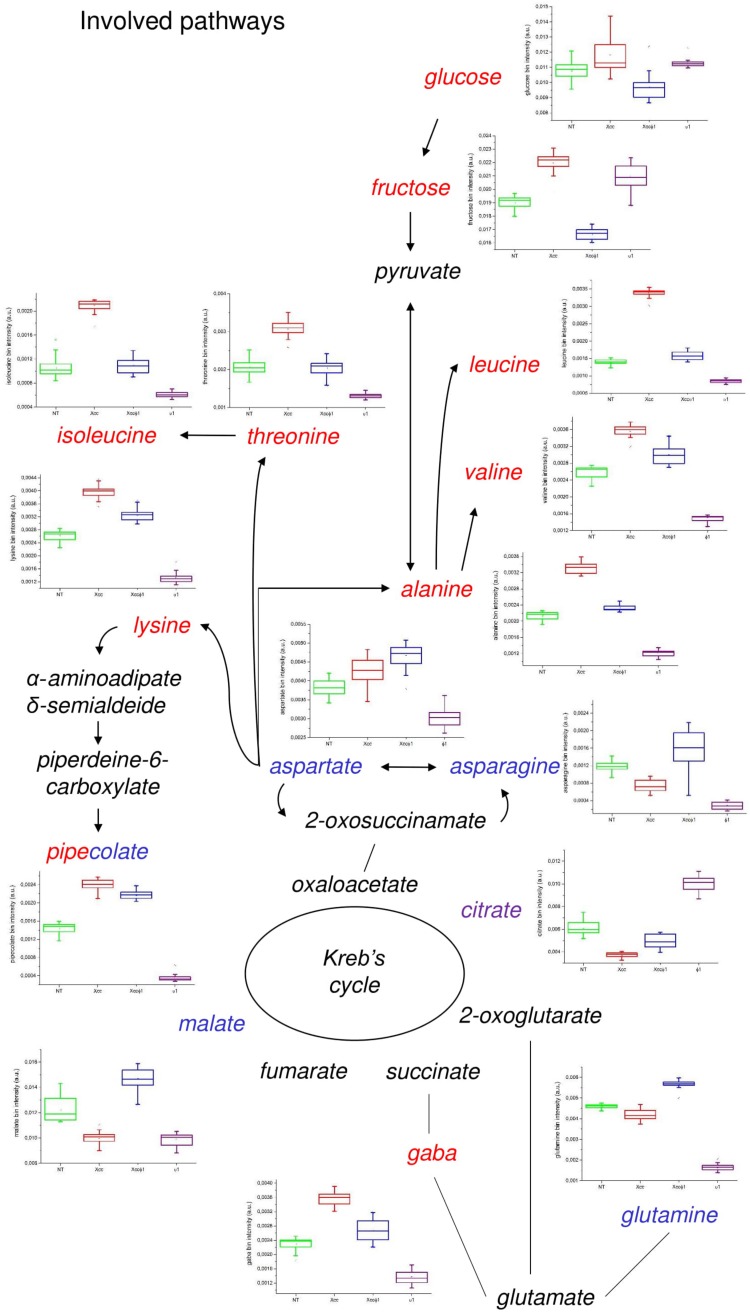
Discriminating metabolites and metabolic pathways observed in the *B. oleracea* var. *gongylodes* metabolome after treatments with NT, *Xcc*, *Xcc* + Xccφ1, and Xccφ1 classes as in Figure 6. Box-and-whisker plots show the variations of the metabolite concentration (green = NT, red = *Xcc*, blue = *Xcc* + Xccφ1, and purple = Xccφ1). The relationships among the metabolites are indicated by lines and arrows. The names of the metabolites are related to the color legend that corresponds to the treatment where they are most accumulated.

The effect on the accumulation of each single metabolite is reported in Figure 7. Specifically, in the *Xcc* group higher levels of branched chain amino acids (valine, leucine, and isoleucine), threonine, lysine, alanine, and GABA (γ-aminobutyric acid) were observed compared to the other groups. Interestingly, there was a lower concentration of these metabolites in the *Xcc* + Xccφ1 group compared to the *Xcc* group. Similarly, the concentrations of glucose and fructose were higher in the *Xcc* group compared to the treatment *Xcc* + Xccφ1.

Pipecolate appeared in relatively high concentrations in both the *Xcc* and the *Xcc* + Xccφ1 groups compared to the other groups. The treatment with both bacterium and phage (*Xcc* + Xccφ1) increased the concentration of aspartate, which is the precursor of lysine, asparagines, and glutamine, compared to all the other samples.

Finally, in the Xccφ1 group, higher levels of citrate and lower concentrations of valine, leucine and isoleucine, threonine, lysine, alanine, GABA and pipecolate were observed compared to all the other treatments.

To the best of our knowledge, this is the first study specifically addressing the changes of metabolic profile occurring in plants infected by pathogenic bacteria and concurrently inoculated with a disease-controlling bacteriophage.

### Expression Profiling of Plant Genes by qPCR

**FIGURE 8:**
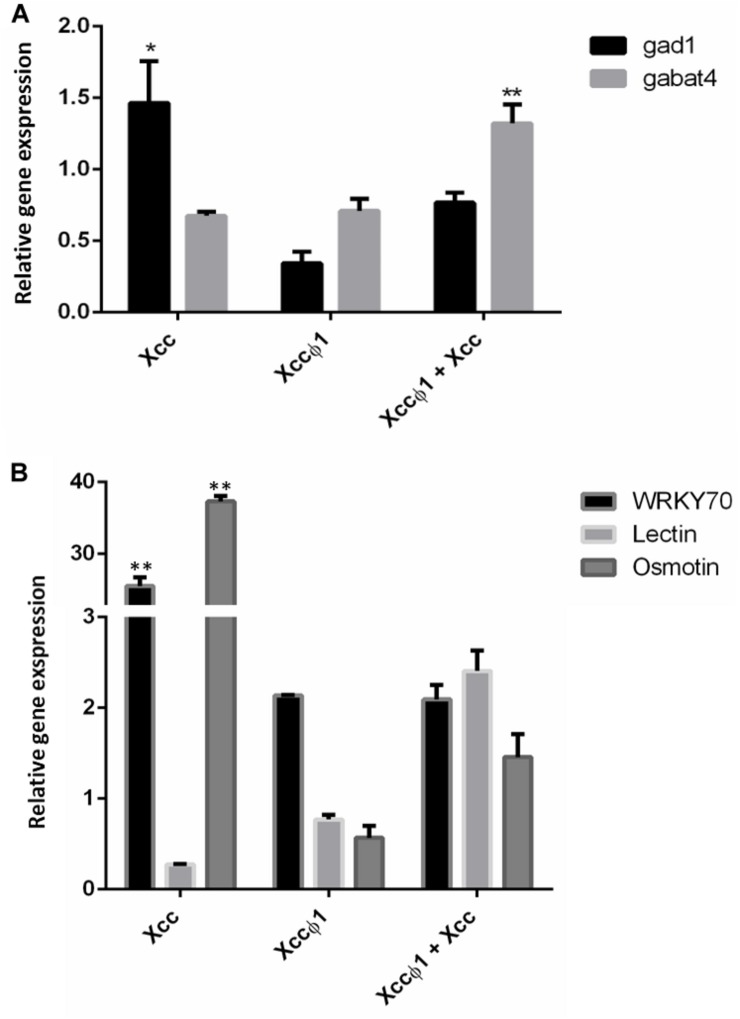
Expression profiling of *B. oleracea* var. *gongylodes* genes by quantitative real time PCR (qPCR). **(A)** Analysis of genes involved in GABA synthesis (Glutamate decarboxylase- 1, GAD1) and degradation (GABA-transaminase 4, GABA-T4). Plant samples were collected at 15 days post inoculation with *Xcc*, *Xcc* + Xccφ1 or Xccφ1. **(B)** Analysis of genes involved in plant disease resistance. Plant samples were collected at 48 h post inoculation with *Xcc*, *Xcc* + Xccφ1 or Xccφ1. WRKY 70: WRKY transcription factor 70. Lectin: legume lectin family protein. Osmotin: osmotin 34. Statistical analysis was performed with Student’s *t*-tests (^∗^ = *p* < 0.05; ^∗∗^ = *p* < 0.001).

Quantitative real time PCR (qPCR) was used as a validation tool to confirm metabolomic data. In particular, the expression of the key genes in GABA synthesis (Gad1) and degradation (GABA-T4) were analyzed 15-days after treatments. As shown in Figure 8A, in *Xcc*-infected plants Gad1 was found to be significantly up-regulated. On the other hand, in infected plants treated with the phage (*Xcc* + Xccφ1), the up-regulation of GABA-T4 was observed. No significant differences were observed in the expression of both genes in plant treated with the phage alone (Xccφ1; Figure 8A).

In addition, the expression profiles of resistance genes WRKY transcription factor 70 (WRKY 70), legume lectin and osmotin 34 were investigated at 48 h post inoculation (hpi; Figure 8B). *Xcc*-infected plants showed a consistent over-expression of WRKY 70 and osmotin genes compared to the control (>25 and >37 Fold Change, respectively). No significant differences were observed in the expression of these genes in plants treated with the phage alone or combined with the bacterium. Similarly, the expression of legume lectin gene resulted to be unaffected by the treatments.

## Discussion

Phage therapy represents a research field with great potential as a new and environmentally sustainable crop protection strategy (Fernández et al., 2018). Several studies have already described the *in vitro* efficacy of bacteriophages against different pathogenic strains of *Xcc* (Weiss et al., 1994; Renu et al., 2017), *Dickeya solani* (Hildebrandt et al., 2015), *Ralstonia solanacearum* (Fujiwara et al., 2011), *X. campestris* pv. *vesicatoria* (Balogh et al., 2003), and *X. axonopodis* pv. *citri* (Iriarte et al., 2007). In all these cases, a high dose of phages combined with antimicrobial molecules was needed to reach a moderate level of disease control, typically up to 20% reduction of symptoms. In a field application, disease symptoms caused by *X. arboricola* pv. *pruni* on peach trees and fruits were reduced by using a bacteriophage (Zaccardelli et al., 1992).

In the present study, we describe the phage Xccφ1, that, applied alone at a MOI of 10, reduces *in vivo* the symptoms of black rot disease by up to 45% (Figure 5).

Disease control requires bacterial biofilm disruption, as demonstrated at least in the case of human pathogens (Fong et al., 2017; Morris et al., 2019). Using CLSM analysis, we found that after 6h of incubation, Xccφ1 disrupts the stability of *Xcc* biofilm (Figure 4). Moreover, we observed the presence of galactose as one of the main components of Xccφ1 particles (Figure 3B). The crystal violet assay highlighted the significant effect of the galactose in reducing the amount of biofilm (Supplementary Figure S1), thus confirming the well documented inhibitory activity of galactose in biofilm formation (Ryu et al., 2016). We therefore suggest that the efficacy of the phage is, at least in part, mediated by phage galactose.

Plant-pathogen interaction causes a drastic metabolic reprogramming, needed to accumulate sugars as *C* source and amino acids as *N* source (Vogel-Adghough et al., 2013; Fagard et al., 2014; Figure 7). Amino acids provide also precursors of secondary metabolites, including a variety of antimicrobial compounds involved in plant defenses (Fagard et al., 2014; Camañes et al., 2015). In line with the above evidence, leaves of *B. oleracea* infected with *Xcc* showed increased levels of glucose, fructose, branched chain amino acids (BCAAs), and lysine, indicating a metabolic transition from photosynthesis to a respiratory metabolism, required to initiate a full defense response (Wishart et al., 2007). This is in accordance with what observed in crucifers infected with compatible or incompatible *Xcc* strains and in *A. thaliana* infected by *P. syringae* pv. *maculicola* (Brauc et al., 2011; Fagard et al., 2014). BCAAs have a role also in human and animal metabolism as modulators of glycolysis and inflammation (Papathanassiu et al., 2017), supporting analogies between plant and animal innate defense mechanisms.

An additional non-protein amino acid, pipecolate, accumulated in *Xcc*-infected and, interestingly, also in *Xcc* + Xccφ1 treated plants. Pipecolate accumulation is involved in the host response to bacterial infection and the establishment of SAR, possibly leading to a long-lasting and broad-spectrum resistance (Wishart et al., 2007; Navarova et al., 2012; Kang et al., 2018). In addition, this compound supports the activation of enhanced pathogen-induced defense responses associated with salicylic acid biosynthesis and priming (Navarova et al., 2012; Hartmann et al., 2017). However, since pipecolate is a common lysine-catabolite, our data suggest that its accumulation at 15 dpi is more related to lysine degradation than to SAR response. This observation is supported by the over-accumulation of the lysine amino acid in plants treated with the bacterium alone or combined with the bacteriophage.

On the other hand, we observed the up-regulation of two resistance-genes (WRKY 70 and osmotin 34) in *Xcc*-infected plants at 48 hpi. The WRKY 70 is considered a key-player in plant responses mediated by salicylic and jasmonic acids and its over-expression is related to the activation of SAR (Li et al., 2017). Furthermore, the *Xcc* + Xccφ1 treatment did not determine effects on the expression of these genes, suggesting that the phage act directly on the bacterium rather than on the activation of plant defense responses.

In plants, the four-carbon non-proteinogenic amino acid γ-aminobutyric acid (GABA) regulates multiple functions: cytosolic pH, osmolarity, cell signaling and reactive oxygen species (ROS) production (Kim et al., 2013a; Batushansky et al., 2014; Hildebrandt et al., 2015). Since it is a molecule synthesized mainly from glutamate and strongly associated with the Krebs cycle, GABA is an important component of the balance between carbon and nitrogen metabolism in plant cells (Michaeli and Fromm, 2015). Interestingly, an increased production of GABA was observed only in *B. oleracea* infected with *Xcc.* Moreover, the key gene involved in the GABA biosynthetic pathway (i.e., GAD1) was over-expressed in *Xcc*-infected plants. This evidence is consistent with metabolomic results. On the other hand, the up-regulation of GABA-T4 could explain the decrease of GABA accumulation in infected plants treated with the phage (*Xcc* + Xccφ1).

In plants treated with both the bacterium and the phage (*Xcc* + Xccφ1) there was an increase in the concentration of primary products of nitrogen assimilation (aspartate and glutamine) and of amino acids normally used as nitrogen storage and transport compounds, such as asparagine (Hildebrandt et al., 2015).

Finally, a significant effect of the phage alone on plant metabolism was observed (Figure 7). Plants treated with Xccφ1, compared to the water control (NT in Figure 7), displayed a general decreased accumulation of amino acids and nitrogen-containing compounds. Interestingly, this effect concerned all of the nine amino acids analyzed, as well as pipecolate, malate and fumarate. On the contrary, citrate accumulation strongly increased, while the level of glucose and fructose were substantially unaffected. Possibly, the presence of the phage alone stimulated the conversion of the amino acid carbon skeleton into precursors/intermediate of the Krebs cycle, in order to support mitochondrial metabolism and the production of ATP (Fujiwara et al., 2011). Coherently, an increased catabolism of amino acids produced a higher level of citrate production compared to control (Figure 7; Batushansky et al., 2014). To the best of our knowledge this is the first time that these effects of a phage on plant metabolism have been demonstrated and we were unable to recover similar data from the literature. In the future it will be interesting to further analyze the reasons why these pathways were activated upon infection with the phage alone. A better understanding of this phenomenon may support an effective application of phages to control plant diseases.

## Conclusion

In conclusion, the *Xcc*-phage interaction discussed here may represent a model to study other combinations of plants with biofilm-producing bacteria, such as olive trees (*Olea europaea* L.) and *Xylella fastidiosa*, with the latter sharing a high genome homology with *Xcc* (Moreira et al., 2005). The resulting knowledge may also be useful in the fight against human pathogens, such as strains of *Pseudomonas aeruginosa*, that form biofilm and are highly resistant to antibiotic therapy (Rasamiravaka et al., 2015).

## Data Availability Statement

The raw data supporting the conclusions of this article will be made available by the authors, without undue reservation, to any qualified researcher.

## Author Contributions

RC have made major contributions to the conception and design of the study. MP, MR, EP, RM, AL, MT, and AM to the acquisition, analysis, or interpretation of the data. MP, AF, AC, GM, AZ, and DP performed the experiments and participated to the interpretation of data. RC, ML, and SW wrote the manuscript. All authors read and approved the final manuscript.

## Conflict of Interest

The authors declare that the research was conducted in the absence of any commercial or financial relationships that could be construed as a potential conflict of interest.

## Footnotes

1 https://www.R-project.org
