# Supplementary material for: Plant Dynamic Metabolic Response to Bacteriophage Treatment After Xanthomonas campestris pv. campestris Infection
Source: Front Microbiol. 2020 Apr 22;11:732. doi: 10.3389/fmicb.2020.00732 (PMC7189621; doi:10.3389/fmicb.2020.00732)
Supplement: Supplementary file 1 [file Data_Sheet_1.docx]

**Figure S1.** Detection of *Xcc* biofilm production using Christal violet assay after 4h of treatment with different concentrations of galactose (gal), as compared to control (ct). Each value is the mean ± SD of 3 independent experiments. *** *p*<0.001. Statistical analysis was performed with Student’s t tests.

*

*

**Figure S2.** Effect of the Xccφ1 phage treatments on *Xcc* disease severity, as measured by the McKinney index, with foliar applications to *B. oleracea* var. *gongylodes*. Different timing of phage and pathogen applications to the plant, using equal concentrations of the phage and bacterium (10^7^ PFU/mL and 10^7^ CFU/mL respectively).

.

*
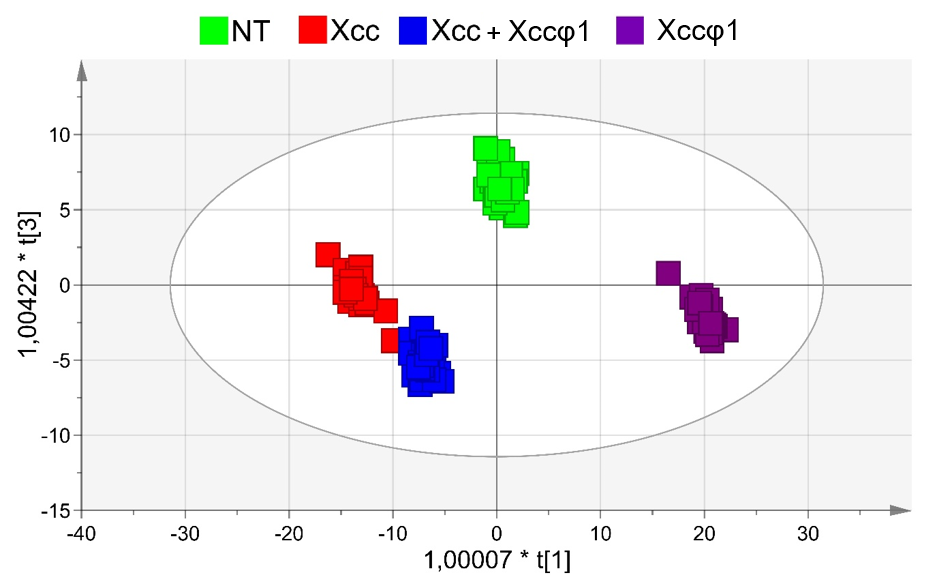
*

**Figure S3.** Score plot t[3]/t[1] showing the projection of the leave extracts NMR spectra onto the third and the first components associated to the OPLS-DA statistical model.


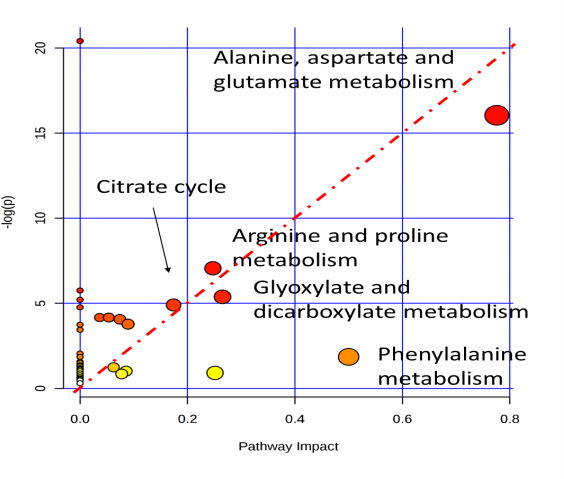


**Figure S4.** Pathway topology and biomarker analysis of *B. oleracea* var. *gongylodes* of discriminating metabolites and processes. Class separation was performed by using Metaboanalyst *4.0.*

**Table S1**. Phage host range determination on different *Xanthomonas* strains from the Plant Pathogenic Bacteria Collection of the Department of Agriculture of the University of Naples Federico II

| Bacteria strains - n° | Host - Crop | Origin | Lytic activity |
| --- | --- | --- | --- |
| *Xc* pv. *campestris* 1 | Kohlrabi - Open field crops | Lazio Region - Italy | + |
| *Xc* pv. *campestris* 3 | Kohlrabi - Open field crops | Campania Region - Italy | + |
| *Xc* pv. *campestris* 1 | Cauliflower - Open field crop | Lazio Region - Italy | + |
| *Xc* pv. *campestris* 2 | Cauliflower - Open field crops | Campania Region - Italy | + |
| *Xc* pv. *campestris* 2 | Cauliflower – Nursery Glasshouses | Campania Region - Italy | + |
| *Xc* pv. *campestris* 1 | Cabbage - Open field crop | Campania Region - Italy | + |
| *Xc* pv. *campestris* 2 | Rocket - Glasshouses | Campania Region - Italy | + |
| *Xc* pv. *vitians* 1 | Lettuce - Open field crop | Campania Region - Italy | - |
| *Xc* pv. *vitians* 1 | Lettuce - Open field crop | Calabria Region - Italy | - |
| *Xc* pv. *incanae* 1 | Stock - Nursery Glasshouse | Campania Region - Italy | - |
| *X hortorum* pv. *pelargonii* 2 | Pelargonium - Nursery Glasshouses | Campania Region - Italy | - |
| *X axonopodis* pv. *phaseoli* 3 | Bean - Open field crops | Basilicata Region - Italy | - |

-, Negative lysis result; +, positive lysis result.

**Table S2**. 1H and 13C chemical shift assignment (δ, ppm) of metabolites found in 1H-TOCSY and 1H- 13HSQC-NMR spectra of B. oleacea var. gongylodes leaves extracts.

| Entry | Metabolite | δ ^1^H | δ ^13^C | Group | Entry | Metabolite | δ^1^H | δ^13^C | Group |  |
| --- | --- | --- | --- | --- | --- | --- | --- | --- | --- | --- |
| 1 | Ile | 0.94  1.01  1.48  1.98 | 11.80  15.40  -  36.60 | δCH_3_  γ'CH_3_  γ'CH  βCH | 19 | Gln | 2.14  2.45  3.77 | 27.13  31.70  55.06 | βCH_2_  γCH_2_  αCH |  |
| 2 | Leu | 0.96  1.71 | 22.70  40.50 | δCH_3_  γCH_2_ | 20 | Succinate | 2.39 | 34.96 | α, βCH_2_ |  |
| 3 | Val | 0.98  1.04  2.28  3.62 | 17.40  18.60  29.70  61.10 | γCH_3_  γ’CH_3_  βCH  αCH | 21 | Malate | 2.40  2.68  4.30 | -  43.43  71.24 | β’CH  βCH  αCH |  |
| 4 | Propionate | 1.05  2.19 | 11.05  31.51 | βCH_3_  αCH_2_ | 22 | Citrate | 2.52  2.65 | 46.47  46.47 | α,γCH  α’,γ’CH |  |
| 5 | Ethanol | 1.19  3.67 | 17.2  56.8 | CH_3_  CH_2_ | 23 | Asp | 2.68  2.79  3.89 | 37.10  -  52.70 | 𝛽CH  𝛽 ’CH  𝛼CH |  |
| 6 | Fucose | 1.27  3.60  3.85  4.12  5.20 | 19.41  75.59  70.91  96.21  96.76 | CH_3_  γCH  εCH  βCH  αCH | 24 | Methyl-guanidine | 2.83 | 38.00 | CH_3_ |  |
| 7 | SFA | 1.31  1.56  2.17 |  | CH_2_ | 25 | Choline | 3.13  3.43  3.96 | 55.00  56.60  68.30 | N-CH_3_  𝛽CH_2_  𝛼CH_2_ |  |
| 8 | Lactate | 1.32  4.11 | 20.76  69.33 | 𝛽CH_3_  𝛼CH | 26 | Ethanolamine | 3.13  3.81 | 43.93  60.57 | -CH_2_-NH_2_  OH-CH_2_- |  |
| 9 | Thr | 1.32  3.60  4.25 | 20.00  61.00  66.60 | 𝛾CH_3_  𝛼CH  𝛽CH | 27 | His | 3.14  3.23  3.98  7.07  7.87 | -  29.15  55.30  117.50  137.17 | 𝛽CH  𝛽 ’ CH  𝛼CH  C4H,ring  C2H,ring |  |
| 10 | Acetoin | 1.37  2.23  4.43 | 19.54  24.94  73.16 | CH_3_-CH-  CH_3_-C=  CH | 28 | Glucose | 3.24  3.40  3.46  3.53  3.73  3.82  3.90  4.63  5.24 | 76.95  72.34  78.57  74.19  63.35  74.13  63.47  98.71  94.93 | C3H  C5H  C6H  C3H  C4H; CH_2_  C6H;CH_2_  CH_2_  C2H  C2H |  |
| 11 | Ala | 1.48  3.79 | 16.80  51.10 | 𝛽CH_3_  𝛼CH | 29 | Betaine | 3.25  3.90 | 55.85  68.64 | CH_2_  CH_3_ |  |
| 12 | Pipecolate | 1.63  1.87  2.21  3.01  3.60 | 29.36  24.55  24.57  46.66  61.92 | 𝛽 CH_2;_  𝛾CH_2;_  𝛿CH_2._  𝛽CH_2;;_𝛾CH_2_ 𝜀CH_2_  𝛽 CH_2_ CH | 30 | myo-Inositol | 3.29  3.54  4.07 | 75.13  73.29  73.10 | C5H  C1H  C2H |  |
| 13 | Lys | 1.65  1.88  3.01 | 26.70  30.20  39.50 | 𝛿CH_2_  𝛽CH_2_ 𝜀CH_2_ | 31 | Methanol | 3.34 | 51.43 | CH_3_ |  |
| 14 | Arg | 1.68  1.92  3.23  3.78 | 24.40  28.00  41.00  54.60 | 𝛾CH_2_  𝛽CH_2_  𝛿CH_2_  𝛼CH | 32 | 𝛽  Galactose | 3.48  3.63-3.65  3.70  3.75  3.93 | 72.90  73.80  76.00  62.00  69.70 | C2H  C3H  C5H  C6H  C4H |  |
| 15 | GABA | 1.88  2.28  3.00 | 24.54  35.17  40.11 | 𝛽CH_2_  𝛼CH_2_  𝛾CH_2_ | 33 | Fructose | 3.50  3.60  3.70-3.73  3.82  3.90 | 66.53  64.73  66.52  70.00  72.37 | C10H_2_  C2H_2_  C10H_2;_ C2H_2_  C3H;C4H  C5H |  |
| 16 | Acetate | 1.92 | 24.07 | CH_3_ | 34 | Sucrose | 3.58  3.77  5.42 | 71.90  73.40  93.00 | G2H  G3H  G1H |  |
| 17 | Pro | 2.01  2.08  2.34  3.33  3.40  4.14 | 23.90  -  29.20  -  46.00  61.10 | 𝛾CH_2_  𝛽 ’ CH  𝛽CH  δ ^’^CH  δCH  𝛼CH | 35 | 𝛼  Galactose | 3.73  3.84  5.26 | 62.20  70.20  93.20 | C6H  C3H  C1H |  |
| 18 | Glu | 2.09  2.34  3.75 | 27.60  34.00  55.20 | 𝛽CH;𝛽 ’CH  𝛾CH_2_  𝛼CH | 36 | Tyr | 6.88  7.18 | 117.00  130.00 | C3, 5H,ring  C2, 6H,ring |  |
| 37 | Fumarate | 6.52 | 136.08 | 𝛼, 𝛽C=C |  |  |  |  |  |  |

**Table S3:** List of genes selected for qPCR analysis. For each one, primer name, sequences and the references were reported.

| **Gene name** | **Primer name** | **Primer sequences 5'-3'** | **Reference** |
| --- | --- | --- | --- |
| Glutamate decarboxylase 1 | Bj_GAD_f | ATGGTGCTCTCTCACGCCGC | Kim et al., 2013 |
|  | Bj_GAD1_r | CTTGGATTACCGTCAAGCATCAACTC |  |
| GABA-transaminase 4 | GABA-T4_f | CGCAAGAAAGAAATCGTATCA | Faës et al., 2015 |
|  | GABA-T4_r | GTGAGGGCAATCTGTGTGT |  |
| WRKY 70 | WRKY_f | TCTGCTCTTGATTCCTTAGAACCCG | Wu et al., 2017 |
|  | WRKY_f | GGTCCAAGTCTTTTCCGACTATCAC |  |
| Legume lectin family protein | Lectin_f | GAAAGCTGGTTACTGGGTTCAGACA | Wu et al., 2017 |
|  | Lectin_r | GCGAGCGTAATGGTAATCCTATTG |  |
| Osmotin 34 | Osmo34_f | GGCTGAGTATGCTTTGAACCAGTTC | Wu et al., 2017 |
|  | Osmo34_r | AGGACACTGTCCGTTTATGTCTG |  |
| Actin_HK | Bj_Actin_f | CCGACCGTATGAGCAAGGAAATC | Kim et al., 2013 |
|  | Bj_Actin_r | TTCCTGTGGACAATGGATGGAC |  |
